# The safety, health, and well-being of healthcare workers during COVID-19: A scoping review

**DOI:** 10.3934/publichealth.2023042

**Published:** 2023-07-21

**Authors:** Abdulqadir J. Nashwan, Rejo G. Mathew, Reni Anil, Nabeel F. Allobaney, Sindhumole Krishnan Nair, Ahmed S. Mohamed, Ahmad A. Abujaber, Abbas Balouchi, Evangelos C. Fradelos

**Affiliations:** 1 Department of Nursing, Hazm Mebaireek General Hospital (HMGH), Hamad Medical Corporation (HMC), Doha, Qatar; 2 Department of Nursing, National Center for Cancer Care and Research (NCCCR), Hamad Medical Corporation (HMC), Doha, Qatar; 3 Department of Nursing Education, National Center for Cancer Care and Research (NCCCR), Hamad Medical Corporation (HMC), Doha, Qatar; 4 Department of Nursing Education, Al-Wakra Hospital (AWH), Hamad Medical Corporation (HMC), Doha, Qatar; 5 Department of Nursing, Iran University of Medical Sciences, Tehran, Iran; 6 Laboratory of Clinical Nursing, Department of Nursing, University of Thessaly, Larissa, Greece

**Keywords:** COVID-19, pandemic, safety, health, well-being, healthcare workers

## Abstract

The outbreak of the COVID-19 pandemic has affected the safety and well-being of healthcare workers. A scoping review was conducted to highlight the impact of COVID-19 on the safety, health, and well-being of healthcare workers and to shed light on the concerns about their perceived safety and support systems. A literature search was conducted in three different databases from December 1, 2019, through July 20, 2022, to find publications that meet the aim of this review. Using search engines, 3087 articles were identified, and after a rigorous assessment by two reviewers, 30 articles were chosen for further analysis. Two themes emerged during the analysis: safety and health and well-being. The primary safety concern of the staff was mostly about contracting COVID-19, infecting family members, and caring for patients with COVID-19. During the pandemic, the health care workers appeared to have anxiety, stress, uncertainty, burnout, and a lack of sleep. Additionally, the review focused on the suggestions of health care providers to improve the safety and well-being of workers through fair organizational policies and practices and timely, individualized mental health care.

## 1. Introduction

The novel coronavirus, which initially surfaced on a limited scale in November 2019, soon spiraled out of control into a global crisis and was declared as a pandemic by the world health organization (WHO) [1]. Frontline healthcare professionals have been impacted by the outbreak, which transitioned into an endemic phase characterized by seasonal or periodic spikes or exacerbations [2]. During the early stages of the pandemic, detailed reporting of COVID-19 sickness and mortality rates among healthcare workers (HCWs) was not readily accessible. In September 2020, six months after the pandemic's commencement, the WHO declared that healthcare professionals accounted for an estimated 14% of the worldwide COVID-19 illness burden [3]. Even today, the lack of a reliable global count of COVID-19 cases among healthcare workers is a major hindrance in getting global data; as of September 21st, 2022, the Centers for Disease Control and Prevention (CDC) COVID Data Tracker recorded 982946 cases among healthcare workers in the United States alone [4]. According to the WHO, between 80000 and 180000 healthcare workers might have perished from COVID-19 globally between January 2020 and May 2021, with a median scenario estimating 115500 fatalities [5]. Frontline HCPs face risks of being exposed to pathogens, working long hours, burnout, stigma, exhaustion, violence, and mental disorders such as fear, anxiety, and depression [6]. COVID-19 infections, inadequate infection prevention and control procedures, workplace safety and health issues, mental health issues, and lack of psychological support for health all impact HCWs' safety, health, and well-being [6]. In this scoping review, a person's perception of his or her ability to handle environmental hazards on a physical, psychological, emotional, and cultural level is referred to as safety, which is demonstrated to influence their personal well-being [7]. According to the founding document of the WHO, “Health is a condition of full physical, mental, and social well-being and not only the absence of sickness or infirmity” [8]. This concept has a crucial implication: health and well-being encompass more than simply the absence of physical or mental diseases and impairments. A person can be said to be in a state of well-being when they are aware of their skills, is able to handle the demands of everyday life, is able to work efficiently, and is able to give back to their community, involving numerous facets including mental and spiritual health [8].

Given the expansive and intricate essence of the subject matter, the selection of a scoping review approach is judicious for a study that aims to delve into healthcare workers' perceptions of safety, health, and wellbeing during the COVID-19 pandemic. This approach, meticulously delineated by Munn et al. [9], caters to an exhaustive exploration of any topic, embracing a spectrum of studies, methodologies, and evidence categories. Notably, it facilitates an extensive literature mapping exercise, thereby enabling the identification of pivotal concepts, the assessment of the existing body of evidence, and the discovery of gaps in the research landscape. Thus, this methodological approach promises a comprehensive understanding of the issue at hand, thereby laying the groundwork for future research trajectories and policy formulation.

On the front lines of the fight against the COVID-19 pandemic, while caring for COVID-19 patients, healthcare professionals are straining and experiencing burnout [10], which causes frustration and posttraumatic stress disorder (PTSD) [11]. Consequently, the primary objective of this review is to delve deeply into healthcare workers' perceptions concerning their safety, health, and well-being in the context of the COVID-19 pandemic. This entails understanding their subjective experiences and unraveling the complex interplay of factors shaping these perceptions. It calls for a consideration of the physical and psychological dimensions of safety and health, as well as the institutional and societal variables that impact their overall well-being. The review is geared towards analyzing how healthcare professionals, who are at the forefront of this global health crisis, perceive their working conditions, protective measures, and access to support and resources. Additionally, it explores the toll this unprecedented crisis has taken on their mental health and personal lives, the coping strategies they have adopted, and their reflections on the adequacy of organizational and governmental responses. Moreover, it seeks to discern any disparities in these perceptions based on roles, geographic locations, and other demographic factors. By compiling and synthesizing this information, this review will shed light on the strengths and weaknesses of current approaches to healthcare worker safety and well-being during the pandemic, thereby informing future research and policy decisions.

## 2. Methods

**Figure 1.**
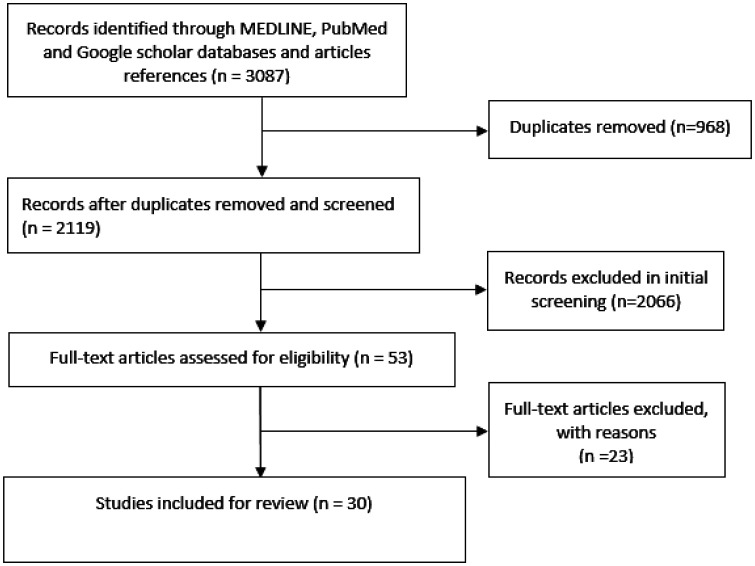
PRISMA-ScR low diagram.

This scoping review was conducted in adherence with the emerging scientific standards for conducting and reporting scoping reviews [12]–[16]. From December 1, 2019, through July 20, 2022, we conducted a literature search within Medline, PubMed, and Google Scholar databases to find publications that discussed the safety, health, and welfare of HCWs during the COVID-19 pandemic. The articles were extracted using the search terms “COVID19 OR covid-19 OR COVID OR novel coronavirus OR coronavirus AND safety OR safe OR wellness OR well-being AND health care OR healthcare worker OR hospital staff OR medical staff OR medical team OR frontline”. We only included the studies that were available as full articles and published in English, without considering design or publication status. Two researchers, R.G.M. and R.A., independently analyzed titles, abstracts, and articles using the same data abstraction form, and selection criteria. Discussion between the two reviewers helped solve data extraction and quality evaluation issues. The exclusion criteria included duplicated articles, non-English articles, and studies that did not concentrate on healthcare personnel and reported study results for other pandemic-related disorders or vaccine efficacies. Data were extracted in an excel format and included the author's name, design, country, population covered ...etc. The article selection that followed the PRISMA-ScR flow diagram [17]–[19] is depicted in Figure 1.

## 3. Results

This scoping review is comprised of 30 studies, of which 25 are quantitative cross-sectional survey studies and five are qualitative phenomenological investigations (Figure 1). Five of the chosen quantitative studies are systematic reviews that include a total of 136 articles. Additionally, there is a systematic review of 10 phenomenological studies in the qualitative investigation. Table 1 displays the nationality coverage of the selected articles, with the majority of studies being from Asia, and more specifically being from China. By including articles from the selected systematic reviews, 22 articles provide data from the middle east, of which five are from Qatar.

Most studies reported on healthcare workers, including physicians, nurses (RNs, LPNs), dentists, psychologists, physiotherapists, pharmacists, radiologists, dieticians, and interns. However, five studies report specifically on nurses, one study covers oncology clinicians, one study covers radiology oncology fellows, and one study discusses senior doctors. In addition, even though most of the study population is from a hospital setting, one study specifically targeted maternal and neonatal health workers, one study targeted the HCWs in quarantine centers, and one study targeted the primary health care setting.

This scoping review primarily focuses on two themes: safety and health and well-being. In this study, as defined by Nissan. Et al, a person's perception of his or her ability to handle environmental hazards on a physical, psychological, emotional, and cultural level is referred to as safety (24). Table 2 exhibits the safety findings from the selected articles. Most studies (60%, n = 18) identified a fear of contracting and spreading the virus to family and friends as the key factor that negatively impacts staff safety perception. Other factors include concerns about the supply and quality of personal protective equipment, increased workload and working hours, lack of proper training and preparedness, social stigma, and physical and psychological violence.

**Table 1.** Summary of the included studies.

**Author (year) reference**	**Type of Study**	**Country**	**Population covered**
Abhiram K (2022)	Cross-sectional survey	Singapore	Health care workers
Allobaney NF (2022)	Cross-sectional, online survey	Qatar	Nurses
Azam F (2022)	Cross-sectional survey	17 countries in the MENA region	oncology clinicians
Bhattacharya PK (2021)	Cross-sectional survey	India, the Middle East, and North America	Health care workers
Biber J (2022)	Cross-sectional survey	USA – Texas and Florida	Health care workers
Chandler-Jeanville S (2021)	Qualitative, Phenomenological	France	Nurses, Nursing Students, and their relatives
Digby R (2021)	Qualitative, Phenomenological	Australia	Health care workers
El Gindi H (2022)	Cross-sectional survey	Alberta, Canada	Health care workers
Galanis P (2021)	A systematic review of Cross-sectional survey studies (16 articles)	China-3, India-1, Japan-1, Turkey-1, Singapore-2, Iran-2, UK-1, Spain-2, Italy-1, USA-2	Nurses
Haidari E (2021)	Cross-sectional survey	USA	Maternal and neonatal HCWs.
Halcomb E (2022)	Cross-sectional survey	Australia	primary health care nurses
Holton S (2021)	Cross-sectional survey	Australia	Health care workers
Jagiasi BG (2021)	Cross-sectional survey	India, the Middle East, and North America	Health care workers
Kader N (2021)	Cross-sectional survey	Qatar	Health care workers
Koontalay A (2021)	A systematic review of qualitative studies (10 articles)	China-2, US-2, UK-2, S Korea-1, Brazil-1, Iran-1, Lebanon-1	Health care workers
Mercado M (2022)	Cross-sectional survey	United States	Health care workers
Muller AE (2020)	A systematic review of Cross-sectional survey studies (59 articles)	China-40, France 2, Germany 2, India 2, Iran 4, Italy 2, Singapore 2, USA 3, Not applicable 2	Health care workers
Nashwan AJ (2021)	Cross-sectional survey	Qatar	Nurses
Nashwan AJ (2022)	Cross-sectional survey	multi-country perspective	Health care workers
Nissan D (2021)	Cross-sectional survey	Israel	Health care workers
Pappa S (2020)	A systematic review of Cross-sectional survey studies (13 articles)	China-12 Singapore-1	Health care workers
Pilar A (2021)	Cross-sectional survey	Canada	Radiation oncology fellows
Ripp J (2020)	Opinion	USA	Health care workers
Salgado (2021)	Cross-sectional survey	United States	Health care workers
Thatrimontrichai A (2021)	A systematic review of Cross-sectional survey studies (32 articles)	**Asia** China-19, S. Korea-1, Saudi-2, Israel-1, Palestine-1, Jordan-1, Turkey-1, Singapore-2, Thailand-1, India-2, Pakistan-1	Health care workers
Tran J (2022)	Qualitative, Phenomenological	Australia	Senior doctors
Villar RC (2021)	Qualitative, Phenomenological	Qatar	Nurses
Vizheh M (2020)	A systematic review of Cross-sectional survey studies (16 articles)	China-9 Italy-1 Spain-1	Health care workers
Wadoo O (2021)	Cross-sectional survey	Qatar	Healthcare workers working in quarantine centers
Wozniak H (2021)	Cross-sectional survey	Switzerland	Health care workers

The theme “health and well-being,” as defined by WHO, (13) investigate the psychological factors that impede an individual from handling the everyday demands of life and work plundering the sense of wellness and order. As identified in Table 2, most studies (73%, n = 22) recognized anxiety and stress as the detrimental factor causing psychological distress, which may have been rooted in the perceived safety concerns. The other findings include depression, sleep disturbances, emotional exhaustion, and work burnout. PTSD [11] and suicidal and harm ideation were also identified as having a negative impact on staff's life.

**Table 2.** The thematic findings from the selected articles.

**Author (year) reference**	**Theme: Safety**	**Theme: Health and well-being**
Abhiram K (2022)	-	* Burnout * Anxiety * Depression
Allobaney NF (2022)	* Fear of contracting/ transmitting the virus * Shortage of PPE * Increased workload	* Decreased self-concept
Azam F (2022)	-	* Stress * Anxiety
Bhattacharya PK (2021)	* Fear of contagion * Getting infected themselves * Insufficient or poor quality of PPE	* Anxiety * Depression * Insomnia
Biber J (2022)	-	* Stress * Anxiety * Sleep-related problems
Chandler-Jeanville S (2021)	* High lethality of this disease * Feared for their relatives' health and their own, * Long working hours	-
Digby R (2021)	* Fear of catching/spreading the virus * PPE * Personal isolation and uncertainty	* Anxiety * Fear * Stress
El Gindi H (2022)	* Fear of contracting the infection * Spreading it to their families * Perceived stigma * Decreased social support	* Stress * Anxiety * Depression
Galanis P (2021)	* Decreased social support * Family or friend infected * Longer working hours * Fear of contagion * Low PPE	* Burnout * Depression * Anxiety * post-traumatic stress disorder * Psychological distress * Sleep disturbances * Insomnia * Fear
Haidari E (2021)	* Patient safety issues * Unprofessional behavior	* Burnout * Emotional exhaustion
Halcomb E (2022)	* Challenges obtaining adequate PPE * Spreading Covid-19 to their family * Fear of contracting Covid-19	* Depression * Anxiety * Stress
Holton S (2021)	* Concerned about passing COVID-19 on to family members * Risk of getting COVID-19 * The possible impact of COVID-19 on pregnancy	-
Jagiasi BG (2021)	* Fear of contagion * Getting infected themselves * Insufficient or poor quality of PPE	* Anxiety * Depression * Insomnia
Kader N (2021)	* Concerned about contracting the disease * Fear of transmitting to family * Fear of social stigma	* Psychological Distress
Koontalay A (2021)	* Insufficient equipment and information * Inadequate PPE * Workload * Need of support from family and friends	* Fear * Anxiety * Depression * Stress * PTSD * Work burnout * Anger
Mercado M (2022)	-	* Stress * Burnout * Insomnia * Anger
Muller AE (2020)	* Exposure to covid-19 patients * Worry about being infected * Worrying about family members being infected	* Anxiety, * Depression, * Sleep problems, * Distress
Nashwan AJ (2021)	-	* Turnover intentions are increased * Stress levels
Nashwan AJ (2022)	* Fear of getting infected * Lack of PPE * Stigma	-
Nissan D (2021)	* Fear of contagion for themselves or loved ones * Caring for unwell colleagues * Inadequate access to personal protective equipment (PPE)	* Traumatic stress * Fatigue * Anxiety * Depression
Pappa S (2020)	* Increased workload * Physical exhaustion * Inadequate PPE * Nosocomial transmission * Loss of social support * Fear of contagion * Medical violence	* Anxiety * Depression * Insomnia * Fear
Pilar A (2021)	* Infection risk * Spreading it to their families * Lack of PPE * Surge of patients (increased workload)	* Burnout * Disengagement * Exhaustion * Anxiety * Stress * Fear * Depression
Ripp J (2020)	* Shortage of medical resources * Limited availability of PPE * Running well over capacity	* Anxiety * Stress * Grief over deaths of fellow clinicians
Salgado (2021)	* Fear of bringing the virus home * Lack of adequate PPE * Lack of proper safety protocols in their place of employment * Long working hours	* Anxiety * Depression * Stress
Thatrimontrichai A (2021)	* Pathogen exposure * Long working hours * Physical and psychological violence with a potential negative impact on patient safety and occupational health * Lack of PPE	* Burnout * Fatigue * Fear * Anxiety * Depression * Occupational stigma * Suicidal and self-harm ideation * PTSD
Tran J (2022)	* Insufficient Quality PP * Lack of PPE training and guidelines * Unsafe working environment * Risk of becoming infected * Concerns about transmission	* Stress * Frustration * Burnout
Villar RC (2021)	* PPE * Fear of contracting the virus * Fear of contagion	* Stress
Vizheh M (2020)	* Workload * Lack of PPE	* Anxiety * Depression * Stress * Insomnia * Distress * Fear
Wadoo O (2021)	* Fear of being infected * Fear of infecting family, * Material resources available for protection, * Vicarious trauma,	* Depression * Psychological Distress
Wozniak H (2021)	* Fear of catching and transmitting COVID-19 * Relatives who had been infected	* Anxiety * Depression * PTSD * Burnout

## 4. Discussion

### 4.1. Safety

The present study reviewed the safety, health, and well-being of HCWs during the pandemic. In this respect, the primary safety concern of the staff was mostly about contracting COVID-19, infecting family members, and caring for patients with COVID-19 [20],[21].

A study by Muller et. al shows that the concern for transferring the COVID-19 infection to a loved one or the event of a loved one's death has a significant influence on the daily stress levels of about one-third of individuals (34.1% and 32.2%, respectively); less than half of the participants showcased fear of dying from COVID-19, while 9.1% reported a “significant” to “severe” impact of the pandemic on their daily life [22]. The knowledge of their own risk of infection and transmission to family members, especially their children and elderly parents in their care, was the primary source of this fear [23].

The healthcare professionals with 1–5 years of experience feared COVID-19 more than those with 6–10 years of experience. Interestingly, despite their high levels of concern, less experienced healthcare practitioners had the best degree of preparedness to treat COVID-19 patients compared to more experienced healthcare providers [24],[25]. Associating age and gender with safety concerns, younger age groups and females showed a more significant proportion of fear and worry [22].

Most healthcare practitioners in the evaluated studies reported being overburdened due to working in crisis circumstances, which impacted them both physically and emotionally due to a lack of time to prepare for the quick rise in new patients [21]. Nurses were affected by this rapid increase in workload and expressed more ‘fear about getting the virus' than doctors and interns. Finally, nurses reported greater mean levels of “fear about infecting family members with the virus” [7].

Concerns were also expressed over the availability and use of personal protection equipment (PPE), redeployment, and their capacity to offer high-quality patient care throughout the pandemic [20]. Fears of utilizing protective gear incorrectly, feeling unequipped to manage patients' nonmedical requirements, and stigmatization as possible infection sources made HCWs feel stressed and vulnerable [23]. Because the frontline medical personnel were compelled to wear PPEs for extended periods, they were noted as unpleasant and were connected with complaints of perspiration, headaches, suffocation, and facial injuries; interestingly, the safety equipment itself turns out to be a stressor for many HCWs [26]. The reviewed studies also demonstrated high levels of stigmatization against HCPs who felt guilt toward their families and friends who could contract the virus from them [25],[27]–[29].

HCWs and their families cited the constant influx of media coverage as a source of stress since they did not know when the COVID-19 outbreak would be contained. The families of HCWs saw them as heroes and were proud of them for putting their lives at risk to care for others, despite weariness and terrible working circumstances; despite their heroism, HCWs were reported to be dismayed and enraged by the governmental authorities' failure to recognize their service [22],[30]. Pregnant employees voiced concerns over the potential effects of COVID-19 on their pregnancy. Few employees had contemplated quitting despite their fears, and good elements of the epidemic were also reported [20].

However, during COVID-19, nurses' intentions to leave were statistically and substantially greater than before COVID-19. Deployment to a COVID-19 facility and working in a COVID-19 facility for more than three months were substantially associated with a greater desire to relocate than those who did not work in a COVID-19 facility [31]–[34].

### 4.2. Health and well-being

Anxiety and stress were recognized as the most prominent components of this theme. The high rate of anxiety and stress could be explained by the fact that COVID-19 appeared unpredictable and potentially lethal, which concerned HCWs and their patients, making them feel helpless; exposure to this uncertainty, combined with media coverage of the events, may have exacerbated it [35],[36].

In addition, the psychological effects of the crisis are experienced not just by frontline respiratory and critical care doctors and nurses, but also by HCWs of other specialties, including surgeons and anesthesiologists. For example, there was a positive correlation between psychological pressure and an intense fear of death with self-efficacy and sleep quality; however, there was a negative correlation with anxiety and stress [37],[38]. A similar trend was seen among oncology professionals in the Middle East and North Africa (MENA) area, who showed a considerable surge in anxiety and stress levels, particularly among females and younger clinicians; however, oncology physicians over 55 years of age working in the public sector reported lower levels of anxiety and stress [39]. The independent risk variables for psychological discomfort include female gender, frontliners, self-COVID-19, and lack of social or emotional support [40],[41].

According to the comprehensive study by Muller et al. [23], the prevalence of mental health distress varied from 7% to 97%, with a median of 37%. Another study supports this evidence, with the prevalence of anxiety and depression in HCWs at 38.6% and 32.2%, respectively. In this research, physicians had the lowest incidence of anxiety and depression, followed by nurses, while other HCW categories had the highest percentage [42]. In another comprehensive study, Pappa et al. [37] observed comparable patterns in the incidence of depression and anxiety among doctors and nurses. Physicians had a lower prevalence of depression (25.4%) than nurses (30.3%), and the same was true for anxiety, which afflicted 21.7% of physicians compared to 25.8% of nurses.

During the pandemic, the health and well-being of HCWs appeared to have worsened, as seen by a rise in coworker burnout and personal emotional weariness. Paradoxically, despite the high rates of burnout, the majority of responders remained optimistic, which may be related to the fact that COVID-19 may provide meaning to their job, playing a role in reducing emotional tiredness in the short term; however, the long-term consequences remain unclear [43]. Numerous senior physicians reported stress, dissatisfaction, and upheavals in their duties and responsibilities at home and the office, resulting in a significant pressure on their work-life balance and burnout. Participants also expressed the feeling of neglect for their safety on the job, and many felt devalued by their employer and the government, with unsatisfactory leadership at all levels [44],[45]. Younger age, a higher level of education, a higher degree, and several social factors, including decreased social support, having a relative or friend diagnosed with COVID-19, low family, and colleague readiness to cope with COVID-19, increased the perceived threat of COVID-19. Longer working time in quarantine areas has been shown to increase HCWs burnout [10],[46],[47].

In the research by Ripp et al. [48] that evaluated sleep quality, about one-third of participants (30.6%) reported having trouble focusing due to poor sleep. In comparison, 67.1% of individuals reported “fair”, “bad” or “very poor” sleep. Other sleep disruptions included having trouble getting asleep (36%) and difficulties remaining asleep/disruptive sleep (50.4%). There were age and gender differences in sleep quality and sleep disruptions, but not occupational risk factors. Younger age groups and females reported lower sleep quality and more frequent sleep disruptions [49].

Infection of coworkers, infection of family members, protective measures, and medical violence were among the primary worries of HCWs in COVID-19-affected regions, according to an examination of variables associated with their psychological challenges [37]. Males experienced more significant depersonalization and lower levels of personal fulfillment, while females reported higher levels of emotional weariness. Mental health concerns were more prevalent among HCWs who worked in a high-risk workplace, such as a COVID-19-certified hospital, a COVID-19 unit, or a critical care unit [10].

## 5. Recommendations

Institutional, state, and national policies should be created or strengthened to alleviate the distress highlighted in this research to regulate rest cycles, leave-taking, child and family care concerns, and workforce allocations. In addition, elements that contribute to or indicate workplace stress, such as shift lengths, patient numbers, leave taken, days off, and sick days, should be tallied in conjunction with a periodic mental health survey.

To eliminate false information, proper communication channels, company-based information technology, and transparency should be promoted. Nurses should be encouraged to rely on official governing bodies for COVID-19 information and to be suspicious of social media-sourced information. Frequent updates regarding the treatment of COVID-19 and accompanying guidelines should be disseminated to nurses.

Staff members require equipment, knowledge, training, clear lines of communication to and from leaders, and encouragement to discuss their experiences. In many instances, peer-to-peer psychological first aid may be beneficial, and sending some staff members for more specialized psychological care may be acceptable. Strategies like virtual coffee mornings, meditation lounges, and an innovative method called “Buddy Up” foster virtual relationships despite physical separation by having groups of two or three persons agree to daily check-ins and monitor one another for stress relief through a phone call, email, or text [50]. Staff working remotely must also be involved, as they may feel high levels of stress and anxiety while not on the front lines. It is crucial that the assistance provided to employees also stresses the enhancement of individual coping skills and resilience. Maintaining contact with supervisors and team members is essential for fostering team spirit, appreciation, respect, and unity.

Providing timely and personalized mental health assistance through hotline teams, the media, or interdisciplinary teams, including mental health specialists, is also crucial for minimizing burnout.

Indeed, the COVID-19 pandemic has underscored the need for proactive, strategic planning for future health crises. Future research should aim to develop effective and adaptable models for managing healthcare worker well-being and mental health in crisis situations. Studies should examine the efficacy of various interventions, including mental health support systems, communication strategies, and contingency planning for healthcare staffing. Additionally, it would be valuable to investigate how lessons learned from COVID-19 can be generalized to other crisis scenarios and how health systems can foster resilience among their workforce. Furthermore, an exploration of the role of technology and remote work capabilities in safeguarding healthcare workers' well-being during crises could offer valuable insights. Ultimately, the goal should be to equip healthcare systems with the knowledge and tools to support their workers effectively, no matter the nature or scale of future crises.

## 6. Limitations

Most studies used in this scoping review were cross-sectional and descriptive in nature, which poses some limitations in generalizability and, more importantly, in their interpretation of causality. This cross-sectional approach with no established baseline prevalence or a control group to compare with makes inferences about the impact of the pandemic on HCWs safety, health, and well-being, in need of supportive evidence from more research. However, including qualitative studies in this review adds to its value of capturing HCWs life experiences and concerns during the pandemic.

Another limitation is the survey format followed by most studies and the recruitment strategy used for its completion. These strategies may have introduced selection bias regarding who completed their surveys and brought an uneven distribution of nurses based on gender and expertise. Moreover, the brief measures included in the surveys do not capture the full extent of stress and work-related outcomes workers may have been experiencing at the time. In addition, many studies were carried out months after the peak of COVID-19, which may have resulted in recall bias. The bulk of investigations being conducted in China and females showing overrepresentation in the review may limit the generalization of the provided results. Furthermore, our decision to include only studies published in English could lead to a selection bias, as it may exclude relevant studies published in other languages. While this decision was made to manage the feasibility of the review, we acknowledge this as a limitation. Finally, when we consider the complexity of this health care scenario, we believe that there might be other concerns present relating HCWs and pandemic beyond the themes considered in this review. This might warrant further studies to address those points.

In most research, pre-existing mental health disorders were not considered, which aggravates the limitation. Individuals with such a history may be more susceptible to relapse or exacerbation of symptoms because of the COVID-19 outbreak's stress [51]. We cannot correctly determine whether a change happened or if the present condition is linked to the pandemic if we do not account for the history of anxiety or sleep problems among participants. Variations in evaluation instruments and cutoffs across studies are another constraint. Several of these studies were done in the same region/country and may have included the same population. On the other hand, several studies have explored the impact of COVID-19 fear and the role of personality traits on the mental health of nurses treating COVID-19 patients. In Japan, for instance, the findings of one study indicated that neuroticism was significantly associated with depression, while both COVID-19 fear and neuroticism significantly predicted anxiety [52]. The study highlights the need for personality-tailored mental health support for nurses. Further studies should be done to explore the role of personality traits as a defense mechanism for dealing with mental health issues.

Despite these constraints, the subgroup analysis of anxiety and depression based on gender, occupational category, and severity gave new insights into possible vulnerabilities. This review contributes to the expanding body of research that recognizes the pressures in combination with work and personal issues and their consequences on employee health and well-being.

## 7. Conclusion

The COVID-19 pandemic is a long-term public health issue, and healthcare professionals are experiencing burnout due to sociodemographic, social, and occupational factors. Measures can be implemented to lessen its effects on nurses, such as screening for mental illness, providing immediate access to mental health care services, giving designated rest periods, and providing social support through hospital support groups. This requires a focus on safe work practices, material resources for patient care and staff protection, ongoing safety training, sufficient personnel, compensation and benefits, and consideration of the emotional requirements of employees.

## Use of AI tools declaration

The authors declare they have not used Artificial Intelligence (AI) tools in the creation of this article.
